# Phenotypes of Management Reasoning Struggle in Postgraduate Medical Education

**DOI:** 10.5334/pme.2960

**Published:** 2026-09-04

**Authors:** Andrew S. Parsons, Matthew Kelleher, James Bowen, Karen M. Warburton, Michael S. Ryan, Walther N. K. A. van Mook, Steven J. Durning

**Affiliations:** 1 Department of Medicine, Division of Hospital Medicine, University of Virginia School of Medicine, Charlottesville, VA, USA; 2 School of Health Professions Education, Maastricht University, Maastricht, The Netherlands; 3 Department of Internal Medicine and Department of Pediatrics, Division of Hospital Medicine, University of Cincinnati College of Medicine/Cincinnati Children’s Hospital Medical Center, Cincinnati, USA; 4 Department of Pediatrics, Division of Hospital Medicine, University of Cincinnati College of Medicine/Cincinnati Children’s Hospital Medical Center, Cincinnati, USA; 5 Department of Medicine, Division of Nephrology, University of Virginia School of Medicine, Charlottesville, Virginia, USA; 6 Department of Pediatrics, University of Virginia, Charlottesville, Virginia, USA; 7 Department of Intensive Care Medicine, Academy for Postgraduate Training, Maastricht University Medical Centre, and School of Health Professions Education, Maastricht University, Maastricht, The Netherlands; 8 Department of Health Professions Education, Center for Health Professions Education, Uniformed Services University of the Health Sciences, Bethesda, Maryland, USA

## Abstract

**Purpose:**

Clinical reasoning struggles are a leading cause of remediation referrals in postgraduate medical education. Most remediation efforts, like much of the clinical reasoning literature, focus on diagnostic reasoning. Errors in management reasoning can have serious consequences for patients, yet no framework exists for recognizing how learners struggle in this domain or for guiding remediation when they do.

**Methods:**

We conducted a multi-institutional qualitative interview study within a constructivist paradigm using the critical incident technique to explore how postgraduate medical trainees struggle with management reasoning in clinical practice, how those struggles are recognized, and how programs respond. Participants were residency program directors and clinical competency committee chairs from U.S. internal medicine and pediatrics programs. Data were analyzed using a systematic cross-case approach to identify phenotypes of management reasoning struggle.

**Results:**

We interviewed 20 program directors and clinical competency committee chairs from 15 programs about 15 trainees remediated for management reasoning difficulties. Cross-case analysis identified four phenotypes: (1) *limited management scripts* (inability to generate or contextualize a plan despite reaching a diagnosis), (2) *can’t change course* (inability to integrate new clinical information into an existing management plan), (3) *impaired task triage and execution* (difficulty prioritizing and coordinating management within a complex clinical environment), and (4) *failure to calibrate* (misjudgment of how much intervention a situation warrants). Contextual (i.e., situational) factors including clinical complexity, patient acuity, diagnostic uncertainty, and reduced supervision increased the likelihood of these phenotypes, and team structures often compensated for struggles before they were recognized. Struggles were frequently misattributed to problems with time management, organization, or communication, and remediation strategies were largely nonspecific across phenotypes.

**Conclusion:**

These four phenotypes offer program leaders and frontline educators an empirically grounded vocabulary for recognizing patterns of management reasoning struggle earlier and designing more targeted support.

## Introduction

Management reasoning, the process underlying decisions about further testing, treatment, monitoring, follow-up, and allocation of limited resources, is now recognized as an important domain of clinical reasoning [1,2]. Errors in management reasoning can have serious consequences for patients, including physical impairment, psychological distress, social disruption, and financial harm [3,4,5]. While clinical reasoning struggles are a leading cause of remediation referrals in postgraduate medical education programs [6,7,8], most remediation efforts, like much of the clinical reasoning literature, focus on diagnostic reasoning [9,10,11]. A previous study of simulated outpatient encounters identified management missteps within single cases, such as failure to follow clinical cues and the absence of shared decision-making [12], but much less is known about the behaviors that characterize management reasoning struggle among postgraduate medical trainees in authentic clinical practice, how these difficulties accumulate across encounters, the clinical contexts in which these struggles become apparent, or how training programs recognize and address these struggles in practice.

Management reasoning competence develops through repeated participation in authentic clinical work [13]. This development depends on the gradual construction of management scripts, pre-compiled conceptual knowledge structures that represent and connect management options with clinician tasks required to carry them out [14,15,16]. Unlike illness scripts, which organize diagnostic knowledge around symptom patterns and disease features [17,18], management scripts organize action by specifying what to do and in what order. Early in training, management scripts are often disease-specific and guideline-based, providing structure but little flexibility in decision-making [13,19]. With repeated exposure to patient care, scripts become more patient and context-sensitive as pattern recognition improves and clinicians grow more able to adapt their reasoning to specific clinical situations [20,21]. This matters because management reasoning rarely has a single correct answer, and decisions require weighing multiple defensible options against the realities of a specific patient and situation [1,2,22].

Script theory describes how clinicians organize management knowledge internally, but situated accounts of cognition suggest that reasoning depends on dynamic interactions between the clinician and the clinical environment [23,24,25]. If reasoning is situated, management reasoning performance should vary across clinical encounters depending on contextual features beyond the case content, a phenomenon called context specificity [26,27]. Because management decisions occur within dynamic clinical environments, difficulties with management reasoning may present as observable behaviors during patient care. The challenge for training programs is distinguishing expected struggle from patterns that warrant formal intervention.

Prior work has proposed strategies for coaching management reasoning [10,11,28], but these approaches are not grounded in empirical descriptions of how management reasoning difficulties manifest among trainees. Without a framework to distinguish management reasoning struggles, remediation efforts may miss the underlying problem and fail to support learners and safeguard patients. To address this gap, we examined cases of postgraduate medical trainees who required remediation for management reasoning difficulties, as described by residency program directors (PDs) and clinical competency committee (CCC) chairs. Specifically, we asked three questions: What behaviors characterize management reasoning struggle in postgraduate medical trainees referred for remediation? In what clinical contexts do these struggles become apparent? How do training programs recognize and remediate these struggles? This study offers a framework for recognizing and naming management reasoning struggle, with the goal of helping training programs intervene more precisely when learners are at risk.

## Methods

Working from a constructivist paradigm [29], we conducted a multi-institutional qualitative study using interviews of residency program leaders. We chose a constructivist approach because management reasoning struggles are not directly observable but are recognized through interpretation of learners’ behaviors within clinical and institutional contexts. This perspective focused our analysis on how program leaders interpreted these difficulties and how those interpretations shaped remediation.

### Participants and setting

Eligible participants were program directors (PDs) or CCC chairs in U.S. internal medicine and pediatrics programs with direct knowledge of a learner requiring remediation for management reasoning difficulties. We used remediation referral as the inclusion threshold because it reflects a program-level judgment that a learner’s performance fell below expectations for their stage of training. Internal medicine and pediatrics were selected because both are high-volume specialties that require sustained management of complex and uncertain clinical conditions across inpatient and outpatient settings, and together they cover a broad range of patient populations. We recruited participants by emailing a convenience sample and used snowball sampling after each interview. Each participant received a $50 electronic gift card.

### Data collection

We used the critical incident technique (CIT), a qualitative data collection approach in which participants describe specific, detailed events that illustrate how a phenomenon occurs in context [30,31,32]. Based on a literature-derived definition of management reasoning [1], participants were asked to identify a learner requiring remediation for management reasoning difficulties. Interviews prompted participants to recount concrete episodes of the learner’s performance, including what occurred, who was involved, how participants made decisions, and what followed. The principal investigator (ASP) piloted the interview guide (Supplemental Digital Material 1) with two PDs who met the inclusion criteria. Early probes were added to parse out knowledge deficits and strengths so that the remainder of the conversation could focus specifically on management reasoning.

Participants were encouraged to review evaluations and remediation documentation before and during the interview to support detailed recall. This approach prioritized cases that were clearly relevant to management reasoning struggles rather than aiming to capture the full distribution of learner performance. We conducted interviews virtually via Zoom (San Jose, CA) by one investigator (ASP) and lasted 60–90 minutes. We audio-recorded interviews, transcribed them verbatim using RevTM (San Francisco, California) [33], and de-identified them before analysis. Interviews continued until four researchers (ASP, MK, JB, KMW) determined through ongoing data analysis that sufficient conceptual depth had been gathered to describe phenotypes.

### Data analysis

**Figure 1:**
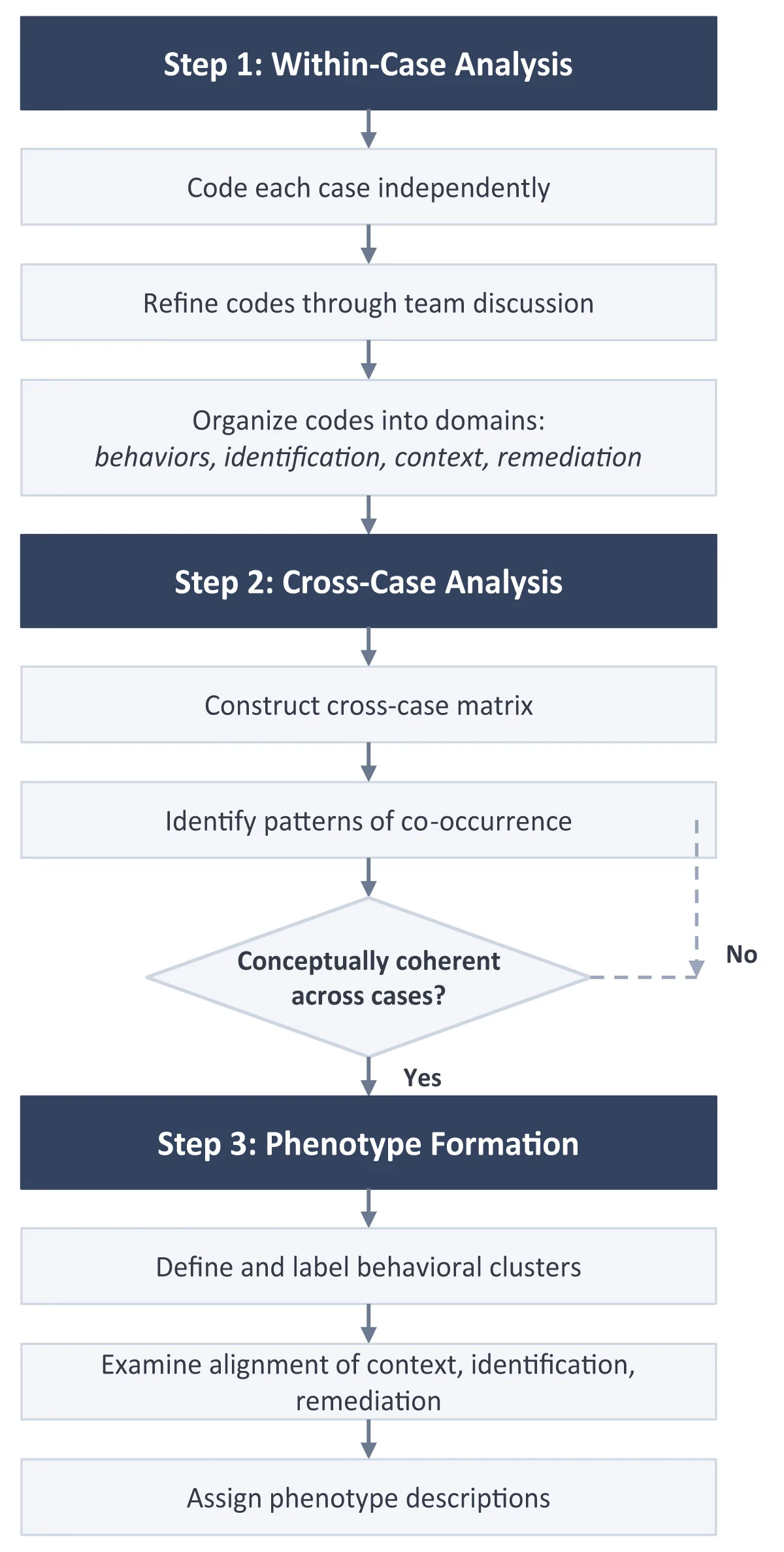
Overview of Data Analysis Process and Phenotype Formation. This flowchart depicts the three-step cross-case analytic process used to identify phenotypes of management reasoning struggle. In Step 1 (Within-Case Analysis), four researchers independently coded each transcript and organized codes into four domains: behaviors of struggle, contextual amplifiers, identification methods, and remediation strategies. In Step 2 (Cross-Case Analysis), a matrix of cases and codes was constructed and examined for patterns of co-occurrence. Conceptually coherent clusters advanced to phenotype formation. In Step 3 (Phenotype Formation), behavioral clusters were labeled and examined for alignment with codes from the other three domains to assign final phenotype descriptions.

We used a cross-case analytic approach described by Miles, Huberman, and Saldaña involving detailed examination of individual cases followed by systematic comparison across cases to identify recurring patterns (i.e., phenotypes) of management reasoning struggle (Figure 1) [34,35]. This approach has been used in health professions education research to explore behavioral and contextual patterns across cases without relying solely on frequency counts [36].

Step 1 (*within-case analysis*): Four researchers (ASP, MK, JB, KMW) independently reviewed each case and wrote analytic memos summarizing key observations and identifying candidate codes as interviews were conducted. Through discussion, we identified and refined case-specific codes. Codes were grouped into identification methods, behaviors of struggle, contextual (i.e., situational) factors that amplified this struggle, and remediation strategies.

Step 2 (*cross-case analysis*): We constructed a matrix with cases as rows and codes as columns, retaining brief supporting text in each cell to preserve context. Beginning with struggling behaviors, the full research team reordered cases and codes to identify clusters. Script theory and situated cognition shaped how we interpreted clusters throughout coding and cross-case comparison. Clusters were retained when they were conceptually coherent. We returned to the transcripts as needed to ensure that clusters were grounded in the case narratives.

Step 3 (*phenotype formation*): After defining behavioral clusters, the research team examined whether contextual amplifiers, identification methods, and remediation strategies aligned with each behavioral cluster, again based on patterns of co-occurrence and conceptual coherence across cases. We labeled these patterns as phenotypes, consistent with the remediation literature [9,10,37], and gave each a descriptive name. Each phenotype was defined by a distinct set of behaviors. A trainee could exhibit behaviors from more than one phenotype, similar to how trainee struggle in other performance domains commonly co-occurs [6], but the phenotypes themselves do not share behaviors. Codes that appeared consistently across clusters without forming patterns were treated as cross-cutting findings. Throughout this process, the team challenged interpretations and ensured consistency with the case data.

### Team and reflexivity

The research team comprised clinician-educators experienced in clinical reasoning education and remediation across medical school and postgraduate medical education (PGME). ASP led the study, participated in coding, and guided phenotype development. His background in qualitative research, clinical reasoning assessment, and coaching informed the focus on observable behaviors and program-relevant implications. MK and KMW, who lead GME remediation at their institutions, and JB, whose research centers on clinical reasoning remediation, participated in coding and helped interpret learner struggles. SJD, MSR, and WNKAM participated in phenotype development and review of coding, drawing on their experience in clinical reasoning scholarship, GME leadership, and qualitative research methods. Throughout the study, the team reflected on how their professional roles might influence interpretation and worked to keep clusters and conclusions grounded in the interview data.

### Ethics

The University of Virginia (UVA) School of Medicine institutional review board reviewed the study and deemed it exempt (IRB #6868).

## Results

**Figure 2:**
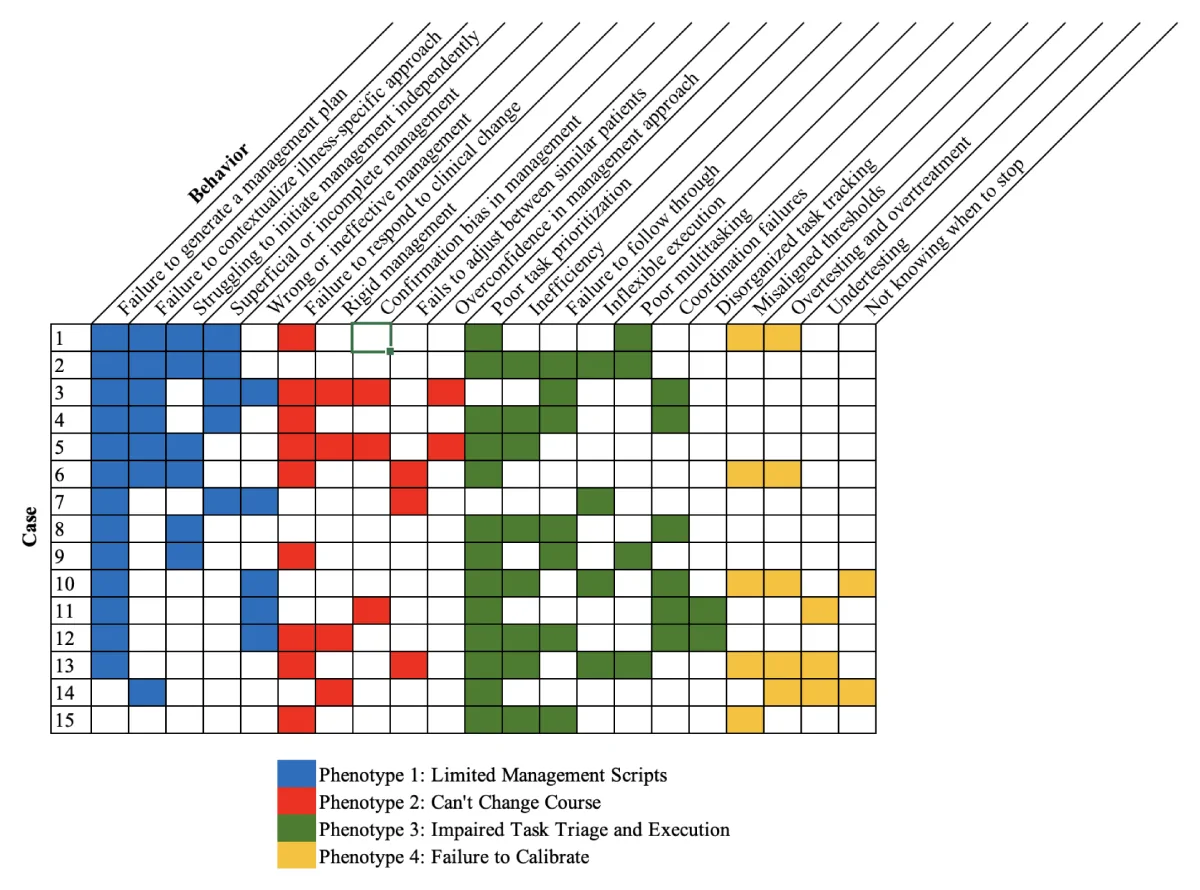
Cross-Case Behavioral Matrix Organized by Phenotype. This matrix displays the 15 learner cases (rows) and behavioral codes (columns) identified through cross-case analysis, organized by the four phenotypes of management reasoning struggle: limited management scripts, can’t change course, impaired task triage and execution, and failure to calibrate. Marked cells indicate the presence of a given behavior within a case, illustrating how co-occurring behavioral codes formed conceptually coherent clusters.

We interviewed 20 residency PDs and CCC chairs from 15 internal medicine and pediatrics programs (i.e., PD and CCC chair were jointly present for five interviews). They described 15 unique learner cases that required remediation for management reasoning difficulties. Learner cases spanned the postgraduate training continuum from early trainees, including interns, to senior trainees in supervisory roles. Cross-case analysis identified four phenotypes of management reasoning difficulty: limited management scripts, can’t change course, impaired task triage and execution, and failure to calibrate. Each phenotype reflects a coherent pattern of behaviors (Figure 2) and, in some cases, associated contextual amplifiers and remediation strategies. Findings that appeared consistently across all four phenotypes are presented as cross-cutting findings (Table 1).

**Table 1:** Cross-cutting Findings: Behaviors, Contextual Amplifiers, Identification Methods, and Remediation Strategies that Appeared Across Phenotypes.


CATEGORY	CODE	DESCRIPTION

Behaviors*	**Freezes under uncertainty**	Trainees stopped acting when the clinical situation lacked a clear answer or established path.

	**Defers to experts**	Trainees handed management decisions to attendings, consultants, or peers rather than forming their own plan.

	**Inability to articulate rationale**	Trainees could not explain the reasoning behind their decisions when asked.

**Contextual Amplifiers**	**High-acuity settings**	Management reasoning struggles were more visible when patients were sicker or more complex.

	**Low-supervision settings**	Struggles became more apparent when attending oversight was reduced.

	**Frequent rotation changes**	Struggles were amplified by transitions between rotations.

	**Frequent attending turnover**	Frequent changes in supervising attendings worsened management reasoning struggles.

**Identification Methods**	**Faculty evaluations**	Written evaluations from frontline supervisors describing concerns about management reasoning.

	**CCC or leadership review**	Concerns identified or escalated during CCC meetings or aggregate review of performance data.

	**Peer trainee feedback**	Concerns raised by fellow trainees, including through chiefs who noted patterns across rotations.

	**Direct observation by program leadership**	PDs or associate PDs personally observed the trainee in the clinical environment.

	**Informal faculty reports**	Supervisors raised concerns through phone calls, side conversations, or emails to program leadership outside of formal evaluation systems.

	**Duty hour review**	Trainees staying significantly later than peers was used as an early signal prompting closer review.

	**Nurse identification**	Bedside nurses noticed and reported concerns about a trainee’s management.

	**Targeted solicited feedback**	Program leadership proactively sought narrative feedback from specific supervisors after an initial concern was raised.

Remediation Strategies**	**Longitudinal one-on-one coaching**	A coach, typically a chief trainee or faculty member, worked with the trainee over time.

	**Clinical reasoning worksheets**	Structured written exercises such as compare-and-contrast tasks and management script templates.

	**Case-based learning**	Guided review of clinical cases with a coach outside of active clinical duties.

	**Schedule modification**	Temporary reassignment to rotations with different acuity levels, supervision structures, or clinical exposures.

	**Increased observation and check-ins**	Chiefs or coaches shadowed trainees through their clinical workflow with frequent structured feedback.

	**Chart-stimulated recall**	One-on-one review of the trainee’s own clinical documentation to examine reasoning behind prior management decisions.

	**External scaffolding**	Structural supports added to the trainee’s workflow, including order read-back systems, pre-rounding checklists, and note templates.


*These behaviors appeared in cases spanning all four phenotypes, suggesting they may represent a common response to management reasoning difficulty regardless of its specific nature. **No site described a formal curriculum for teaching management reasoning.

### Phenotype 1: Limited Management Scripts

PDs described trainees who could gather clinical data and arrive at a diagnosis but could not reliably convert it into a management plan. There was a spectrum of severity. At the most extreme, some trainees were entirely unable to generate a plan:

“He was good at coming up with a differential, but to actually decide what you are going to do, it seemed like that was a real challenge” (Case 8).

“During his plan on rounds, he really didn’t generate a plan. He said we’ll continue what we’re doing” (Case 6).

Others could implement a plan once given one, but could not produce it independently:

“Once a plan was given to him, he was much better at implementing that plan than when he didn’t have the plan” (Case 2).

In some cases, providing the diagnosis appeared to unlock a management plan that the trainee could not generate independently. These trainees had the management knowledge, but they stalled at the step from diagnosis to plan. In other cases, trainees generated plans, but they were internally inconsistent with their own clinical reasoning:

“He would indicate that he thought that the patient was hypovolemic, but then his plan was to continue the diuresis” (Case 12).

Less extreme presentations were also described. Some trainees could generate plans for most patients but struggled when asked to tailor those plans to the specific patient or clinical situation:

“His thinking has been much more black and white. This patient has pancreatitis; they need a CT scan. That’s what we do for patients that have pancreatitis and it’s not going to take into account as many of the contextual factors that may make that a great idea or not as good of an idea” (Case 2).

Others could generate and contextualize plans but lacked the depth the clinical situation warranted:

“They were often too superficial, and they didn’t seem to recognize that…they probably test quite well but would just sort of quick answers, superficial thinking, let’s move on, boom, boom, boom, boom, boom” (Case 3).

These struggles were amplified by clinical complexity and undifferentiated presentations. As the number of active problems increased, the fragility of these trainees’ management scripts became more apparent:

“Failure to balance competing active problems or failure to reconcile multiple problems into larger pictures. And so as you stacked organ system failure or multi disease processes at the same time, I think that’s where things started to fall apart” (Case 5).

### Phenotype 2: Can’t Change Course

PDs described trainees who developed an initial management plan but did not revise it as the clinical picture evolved. At the most consequential end, trainees failed to respond even to urgent clinical changes despite direct prompting:

“A patient had a rising lactate and worsening tachycardia but was admitted to the floor. And despite repeated prodding from nursing about concern for stability, she would not adapt” (Case 3).

This was not limited to high-acuity situations. Routine transitions that required any shift in approach could expose the same difficulty:

“This was the plan, we did the plan, now we’re onto something new and you have to change and interpret. Now that shift is harder for her” (Case 14).

Some PDs described inflexibility in how trainees held their plans regardless of what new information emerged. For some, this was a general rigidity, an inability to entertain that the plan might need to change at all:

“Some people are so rigid, and so I think it’s like a one-track mind. You put the thing in the track and that’s what he can think” (Case 13).

“She was going to try to squeeze the plan into that bucket even though it didn’t really fit” (Case 3).

For others, the inflexibility was more specific. Trainees appeared to filter out information that contradicted their existing plan rather than updating the plan:

“If it didn’t fit his initial plan, it might be there, but he wasn’t going to go look for it and maybe he didn’t even notice it. I don’t know. That’s the way it felt. It was like out of sight, out of mind if it’s not, wasn’t his plan” (Case 3).

A related pattern was applying the same management approach across patients with the same diagnosis without accounting for differences in clinical circumstances. These trainees could generate a plan, but applied it with the same rigidity that characterized the other behaviors in this phenotype:

“Her schema is, I’ve seen so many people with anemia and GI bleed, and we usually get GI involved. That was kind of her first thought without being able to put together the bigger picture” (Case 1).

“Pneumonia, pneumonia, pneumonia, same, same, same. Oh, this one requires something different…that is harder sometimes for her to pick up on” (Case 14).

In some cases, trainees held to their plans with a certainty that exceeded what the clinical situation warranted:

“Just headed down the wrong path and determined to stay on it” (Case 3).

These behaviors were most apparent in settings that required ongoing reassessment rather than adherence to established protocols. PDs noted that the same trainees often performed adequately in algorithmic or protocol-driven settings and that difficulties became apparent when clinical management required deviation from a predetermined pathway. One PD said evaluations:

“didn’t have as much signal in more of the formulaic cookbook areas of our cardiology rotations but that the broader, less focused differentials of critical care, general medicine, and at times oncology were places where some of these issues arose more” (Case 5).

### Phenotype 3: Impaired Task Triage and Execution

PDs described trainees who struggled to translate an appropriate management plan into completed work. A central behavior was not knowing which tasks required attention first. These trainees could not determine which patients or tasks required immediate attention and which could wait:

“Prioritization of what needs to happen for the patient is not something he’s able to generate on his own, which results in a lack of organization of the flow of the day. So, an example…maybe he wouldn’t go see his sickest patient or his child who’s ready to go home first. He might just go in bed order even though he only has 15 minutes to see all his patients” (Case 6).

A related behavior was continuing to execute tasks in a fixed sequence even when the situation called for reprioritization:

“I do the next task on the list. I don’t think about, this is actually not the right task to do. Next, I need to do this other thing…we’ve had potassium of six on it in a similar instance to this versus, well, I’m finishing my notes” (Case 8).

This difficulty with prioritization became most visible when demands accumulated beyond a threshold that trainees could manage. Rather than slowing down or reorganizing, trainees appeared to lose the ability to order their response at all:

“When there are a lot of things happening at the same time, it seems like a struggle to process what has to happen first, second, third; it’s a task prioritization problem…we’ve done simulations with her before and so I’ve watched this happen if you’re doing okay, you’re doing okay. And then you get to a point where there’s too much input and then you can’t organize your tasks” (Case 14).

“When you focus him on one task, he does it pretty well. Now do it all in one patient all at one time, can’t do it” (Case 13).

The consequence of these behaviors was that work either took longer than expected or did not get done at all. Program directors described initially framing this as a time management or organizational problem before recognizing it differently:

“He falls into that category of really hard time task switching and can’t organize their thoughts, spin their wheels all day long, they cannot filter and prioritize the information that’s coming to them. So, it takes them longer to do everything” (Case 1).

“His morning pre rounds were very time consuming. So, he would be late to start rounds because he would still be working on those discharge summaries” (Case 4).

In other cases, agreed-upon tasks were simply not completed:

“Did he not believe that was a good plan, did he not remember that that was the plan, or did he remember that that was the plan and then just forget to implement it with the orders” (Case 12).

Two additional observed behaviors accompanied these patterns. Some trainees lacked a reliable system for tracking what had and had not been done:

“He’d have his list and write notes to himself on his list, but not in any kind of form or order. So, he’d shuffle between papers when he is trying to figure out whether something had been done or whether he called this consultant or that consultant” (Case 4).

In other cases, the team quietly reorganized around the struggling trainee before the difficulty was formally recognized:

“His co-interns were noted to be signing up to each other, helping each other with tasks and not asking him to help with anything because he lacked the ability to sort of manage his own tasks” (Case 6).

These behaviors were most apparent in high-volume inpatient settings. Program directors noted that the same trainees functioned better in outpatient environments where the pace was steadier and fewer demands competed for attention at the same time.

### Phenotype 4: Failure to Calibrate

This phenotype reflected difficulty determining how much management a clinical situation warranted. Trainees in these cases were not making management decisions that were categorically wrong. Instead, they were miscalibrated - doing too much in some situations, too little in others, and sometimes both within the same clinical encounter. Some over-responded:

“He identifies risks and then heightens them. He gets the wrong proportion of that risk and we’re all like, I wouldn’t worry too much about that. And he’s spending six minutes telling you why we should” (Case 13).

For others, this manifested as a broad and indiscriminate approach to testing and treatment:

“She wanted to work up every single thing. And then really particularly…kind of that allocation of resources, she wanted to treat everybody for everything. She wanted to refer every patient to every specialist” (Case 10).

“She tends to over test and over treat. She errs on the side of caution more often she wants more testing or more, she wants more information” (Case 14).

Overresponse and underresponse were not mutually exclusive. The same trainee could exhibit both:

“I think he’s at risk of both over and under ordering” (Case 6).

“When they get down these rabbit holes…they are going to order tests that are unnecessary and not order the tests that are necessary” (Case 13).

For some trainees, the miscalibration appeared specifically as an inability to stop once a reasonable management course had already been established, continuing to pursue additional data or interventions beyond what the situation warranted:

“She would go on these deep, deep, deep dives into people’s records and find all these things and have, if we were a patient that I was seeing in clinic, I probably would’ve addressed two or three things and she had nine things that she wanted to address for every patient, so she would get increasingly far behind” (Case 10).

The same PD summarized this as an:

“overall discomfort with letting good enough be.”

These behaviors worsened in the context of diagnostic uncertainty. In at least one case, a medical error early in training appeared to have deepened an existing tendency toward overcaution. Clinical experience can reinforce these patterns as well as expose them.

## Discussion

We describe four phenotypes of management reasoning struggle grounded in real clinical settings. Each phenotype reflects a distinct behavioral pattern. Struggles were often misattributed to performance domains more familiar to program leaders such as time management or communication, and team structures frequently compensated for trainee performance struggles before programs formally recognized them. Remediation was largely nonspecific to the pattern of struggle.

### The Phenotypes of Management Reasoning Struggle

Management scripts, the conceptual knowledge structures of management options that help organize action, provide a useful lens for understanding the first phenotype [14,15,16]. Trainees with limited management scripts (i.e., phenotype 1) struggled to generate and structure a coherent plan despite gathering relevant data and arriving at a reasonable diagnosis. This pattern parallels the concept of dispersed knowledge organization in diagnostic reasoning, in which relevant knowledge exists but is not structured to support retrieval and application when needed [38,39]. Phenotype 2 (“can’t change course”) reflects a different kind of breakdown. These postgraduate medical trainees chose an initial plan but could not integrate new clinical information as it became available. This pattern points to a limitation in cognitive flexibility, not knowledge organization [20,39]. This pattern is consistent with limits in adaptive expertise, where prior knowledge is applied without sufficient adjustment to changing circumstances [20]. This vulnerability appears to cut across diagnostic and management reasoning, which has implications for how programs design remediation. The behaviors in phenotype 2 are also consistent with well-described cognitive biases, including anchoring bias and confirmation bias, suggesting these diagnostic reasoning patterns extend to the management domain [40]. Clinician-educators who recognize this connection may find the literature on metacognition helpful to support trainees who fit this phenotype [41,42].

Trainees in phenotypes 3 (“impaired task triage and execution”) and 4 (“failure to calibrate”) reflect breakdowns in both cognitive processing and how trainees enact management reasoning within the health care system. Trainees in phenotype 3 often identified reasonable management options but struggled to coordinate and implement them within a complex clinical environment. Ecological and distributed accounts of cognition help explain this finding: reasoning depends on recognizing and using the supports available in the environment, including team members and workflow structures [43,44,45,46]. In some cases, co-trainees or senior trainees had stopped including the trainee in shared work and were quietly completing tasks themselves, which meant the breakdown in execution was masked by the very team structures that distributed cognition accounts predict would support reasoning.

Phenotype 4 is the most contextually sensitive of the four. Prior work has shown that trainees experience management reasoning as an ongoing negotiation of risk and uncertainty [13], and trainees in this phenotype struggled with that negotiation and misjudged how much intervention a situation warranted. Threshold models of clinical decision-making suggest this reflects disruption in analytic risk estimation, the deliberate weighing of disease likelihood against the benefits and harms of acting [47,48,49]. Individual risk tolerance and prior clinical experience also impact how clinicians appraise risk in practice [50,51]. Context specificity offers another explanation. Clinical reasoning performance varies from one encounter to the next for reasons beyond the clinical content [52,53], and recent work shows management reasoning behaves the same way [26]. Trainees whose judgment has been calibrated in a narrow range of clinical contexts may struggle when those contextual features shift, which suggests that exposure to varied clinical environments is critical to developing management reasoning.

### Implications for Educators to Support Struggling Learners

These phenotypes reflect patterns of struggle, not the normal development of management reasoning. They differ from it in severity and timing. Most trainees in our sample showed features of more than one phenotype, which is consistent with the broader remediation literature, where trainee struggles commonly overlap and effective interventions focus on one problem at a time [6,11,54]. There is likely also overlap between the behaviors in each phenotype and those of learners early in training that do not require formal remediation. For example, our current understanding of management reasoning suggests that novice trainees may generate management plans that are less developed than the situation requires and adapt them slowly as new information appears [13]. What distinguished trainees in our study was the persistence and degree of struggle that led to the need for remediation. We view remediation as the intensification of developmental support along a continuum. That continuum runs from the individualized coaching commonly provided to more novice trainees to the remediation reserved for trainees who remain unable or unwilling to change their behavior despite extensive and prolonged educational support.

The phenotypes may also be distributed unevenly across postgraduate training. Because scripts become more nuanced and context-specific with experience, limited management scripts (phenotype 1) may be most prominent early in training. Failure to calibrate (phenotype 4) may become most apparent as trainees take on greater independent responsibility for risk decisions. Testing this prediction would require analyzing management reasoning behaviors across a full cohort rather than only trainees referred for remediation, because our sample cannot separate a developmental trajectory from the point at which struggle draws formal attention.

Earlier recognition is an important predictor of successful remediation [55,56], and having a specific phenotype to point to, rather than a *vague sense that something is wrong*, may help programs move more efficiently from recognition to intervention. A phenotype-informed approach can also support more targeted intervention, both through structured remediation and real-time feedback in the clinical environment. A trainee who generates reasonable plans but cannot adapt them needs remediation that surfaces the reasoning behind plan revision. A trainee who cannot execute management tasks in a team-based environment needs support that addresses workflow and coordination. Aligning remediation strategies with these patterns of struggle may allow educators to tailor feedback and learning experiences more precisely to the learner’s needs.

Programs may also consider the cross-cutting findings in Table 1 as an earlier pathway to recognition. The behaviors listed there appeared across all four phenotypes and may be detectable before a specific pattern has fully formed, and they give frontline assessors an early signal that management reasoning warrants closer attention. The contextual amplifiers are similarly actionable. Attending turnover and team structure are modifiable, and supervisors who know that diagnostic uncertainty and reduced supervision reliably surface these struggles can watch more deliberately when those conditions are present. Faculty development efforts that help supervisors recognize both these behavioral signals and contextual triggers could enable early developmental support, preventing transient learning challenges from becoming entrenched and ultimately requiring remediation.

## Limitations

Our participants were PDs and CCC chairs from internal medicine and pediatrics, and their perspectives reflect how management reasoning struggles are perceived by those in supervisory roles. Learner and frontline faculty perspectives may differ. The use of CIT produced detailed case narratives, but participants’ recall was retrospective and potentially shaped by the outcome of the remediation process. These cases reflect learners whose difficulties were significant enough to reach formal leadership attention. Milder or earlier forms of management reasoning struggle may be underrepresented, as the same behaviors likely appear in subtler form among earlier trainees whose management scripts are still forming. The number of phenotypes we identified are specific to patterns present in this qualitative sample and may not represent the full spectrum of management reasoning struggles encountered across postgraduate training programs.

Given our use of a convenience sample, including a snowball approach, educators who are more engaged in remediation or more familiar with management reasoning may have been more likely to participate. Finally, although we drew on programs across multiple institutions, our sample was limited to two specialties and U.S.-based training programs, and the findings may not transfer to other disciplines or settings.

## Conclusions

In this study, we explored how postgraduate medical trainees struggle with management reasoning and identified four phenotypes of difficulty, along with cross-cutting behaviors, contextual factors, and patterns of identification and remediation. These phenotypes offer program leaders and frontline educators an empirically grounded vocabulary for recognizing management reasoning struggle earlier and designing more targeted support. Future work should examine whether phenotype-informed approaches to remediation improve trainee outcomes and whether these phenotypes can guide developmental support before remediation is warranted.

## Disclaimer

The opinions and assertions expressed herein are those of the author(s) and do not necessarily reflect the official policy or position of the Uniformed Services University of the Health Sciences or the US Department of War.

## Previous Presentations

This work was presented at the International Association for Health Professions Educators (AMEE) annual meeting 2025 as a research paper in Barcelona, Spain.

## Additional File

The additional file for this article can be found as follows:

10.5334/pme.2960.s1 Supplemental File 1. Interview Guide.
