# Supplementary material for: Phenotypes of Management Reasoning Struggle in Postgraduate Medical Education
Source: Perspect Med Educ. 2026 Sep 4;15(1):718–31. doi: 10.5334/pme.2960 (PMC13544060; doi:10.5334/pme.2960)
Supplement: Supplementary file 1 [file pme-15-1-2960-s1.pdf]

Supplemental File 1 – Interview Guide

Welcome:

"Hello, thank you for taking the time to participate in this study. We are interested in exploring the individual behaviors and situational factors contributing to management reasoning struggles among graduate medical learners who require remediation. As described in a prior email, our focus will be on discussing one specific learner from your program who struggled with management reasoning. For purposes of this study, remediation is defined as any program-directed intervention to improve clinical performance above and beyond usual practice. I will ask a series of questions to better understand your experiences and perceptions. Please feel free to reference evaluations, notes, or clinical competency committee reports specific to this learner.

At the end of our discussion, I will also ask you a couple questions to gather background information on you and your role with graduate medical learners. This interview will be audio recorded and transcribed. This session will last approximately 60 minutes.

We will do our best to keep your information confidential. Avoid using the learners name at all times. Only the study researchers will listen to the contents of the tape. You are welcome to decline to answer any of the questions or stop the interview at any time.

Please verbally indicate whether or not you would like to participate in this study.

Let's get started!"

I will now provide a definition from the literature to guide our discussion. Management reasoning has been defined as "the process of making decisions about patient management including choices about treatment, follow-up visits, further testing, and allocation of limited resources. It is, in essence, the part of clinical reasoning that proceeds from the working diagnosis."

Overall

1. **With specific focus on management reasoning, please describe a case of a graduate medical learner in your program who struggled and required remediation?**

Potential probes:

- How did this come to your attention? From who and based on what (i.e. specific assessments?) At what time point was the learner identified? What was the "chief concern" about the learner?
- What was said/documented by this learner's clinical supervisor, colleagues, chief residents, program leaders, CCC and/or remediation coaches?
- How would you describe this learner's fact-based recall/knowledge? What do previous standardized tests suggest?

Behaviors and Performance Deficits

2. **What specific behaviors indicated that this learner was struggling with management reasoning?**

Potential probe:

- How did these behaviors manifest in clinical practice/impact patient care? Give examples (rounds, presentations, other?). Aneqdotes encouraged.
- If you were to frame this learner's struggles in the form of a chief concern, what would that be?

<u>Contextual Factors</u>

3. **What situational factors do you believe contributed to the learner's struggle with management reasoning? For example, somethings that may come to mind include clinical setting, patient type, and workload.**

Potential probes:

- What was the context of this struggle (e.g., clinical setting, patient type, specific circumstances)?
- How did the environment (e.g., clinical workload, EHR use, identification and management of system preferences and constraints, interproffesional team dynamics) impact the learner's performance?
- How about triage/discharge/monitoring/change in patient condition/urgency?
- How did patient circumstances (e.g., complexity, prioritization of patient preferences, shared decision-making, communication more broadly) influence the learner's performance?
- How did the learner navigate diagnostic uncertainty, consider testing and treatment thresholds, and/or incorporate estimates of risk into management decisions?

<u>Remediation</u>

4. **What strategies were employed to remediate this learner's management reasoning struggles?**

Potential probes:

- Which strategies were most effective?
- What barriers did you encounter in the remediation process specific to this learner?

<u>Learner Background</u>

5. **Are there gaps in education or training that you think contributed to this learner's management reasoning struggles? If so, please describe.**

<u>Program Education</u>

6. **How do you teach management reasoning in your program, formally or informally?**

Reflection

7. **Is there anything that I haven't raised today that you think is important regarding your conceptualization of how graduate medical learners struggle with management reasoning?**

Potential probes:

- Any other comments from the documents you have available?

Background Information

8. **What is your medical specialty and current role in medical education?**

9. **How many years have you been involved in supervising and evaluating graduate medical learners?**

Thank you for your time and insights.
